# The Nutritional and Micronutrient Status of Urban Schoolchildren with Moderate Anemia is Better than in a Rural Area in Kenya

**DOI:** 10.3390/nu12010207

**Published:** 2020-01-13

**Authors:** Esther Charlotte Evang, Tsige-Yohannes Habte, Willis Omondi Owino, Michael Bernhardt Krawinkel

**Affiliations:** 1 Institute of Nutritional Sciences, Justus-Liebig-University Giessen, Wilhelmstr. 20, D-35392 Giessen, Germany; esther.c.evang@ernaehrung.uni-giessen.de (E.C.E.); tsige-yohannes.habte@ernaehrung.uni-giessen.de (T.-Y.H.); 2 School of Food and Nutritional Sciences, Jomo Kenyatta University of Agriculture and Technology, Juja, Nairobi 62000-00200, Kenya; willis@agr.jkuat.ac.ke

**Keywords:** nutritional status, micronutrients, dietary intake, urban, rural, schoolchildren

## Abstract

Low diet quality is a driver of general and micronutrient malnutrition in urban and rural areas. The objective was to compare malnutrition and micronutrient deficiencies linked to dietary intake among urban and rural schoolchildren from food insecure settings in Kenya. The cross-sectional study was conducted among urban and rural schoolchildren aged 7–9 years. Height and weight were measured, venous blood samples were assessed and data on dietary intake was collected. After screening out children with hemoglobin >12.2 g/dL and moderate or severe undernutrition, a total of 36 urban and 35 rural children participated. The prevalence of moderate underweight, wasting, and stunting were lower in urban than in rural children, with significant differences in median *z*-scores for underweight (*p* < 0.001) and wasting (*p* < 0.001). Significantly higher values for serum ferritin (*p* = 0.012) and zinc (*p* < 0.001) were found in urban children. Yet, the median adequacy ratios were higher for vitamin C (*p* = 0.045), iron (*p* = 0.003), and zinc (*p* = 0.003) in rural than in urban children. General nutritional, iron, and zinc status were significantly better in slightly anemic urban children than in rural ones. Improving the nutrition of schoolchildren in urban and rural settings requires different dietary approaches.

## 1. Introduction

Adequate nutrient and energy intake through diverse diets are important drivers for healthy child development. In sub-Saharan Africa, diverse diets contribute to overall nutritional adequacy [1,2] and are associated with a good nutritional status [3]. Monotonous, cereal-based diets with low nutrient density are often linked to a lack of access to vegetables, fruits, and animal-sourced food (ASF), causing deficiencies in essential micronutrients [4]. Often, these deficiencies are not apparent, hence they are also called hidden hunger [5].

Living environment, access to clean water, sanitary facilities, health facilities, and food differs between urban and rural settings in Kenya. Food consumers in urban resource poor setting are primarily depended on the market supply, while people in rural settings mainly depend on self-produced agricultural commodities. In urban resource poor settings, the most available and affordable diets are often unhealthy [6]. Likewise, typical street foods in resource poor settings in Nairobi tend to be high in calories and low micronutrient content [7]. In rural Kenyan settings, access to these street foods is limited. The supply of vegetables (kales, tomatoes, onions) in Nairobi is fairly constant across the year [8]. Nevertheless, the poorest people in urban areas tend to be the lowest consumers of fruit and vegetables, which already accounts for 17.1% for vegetables and 6.5% for fruits on their share of food expenditure [9]. In rural settings, the consumption of vegetables varies by season [10].

Children from households affected by food insecurity are particularly vulnerable to micronutrient deficiencies. These do not allow them to reach their full physical and cognitive potential, and their educational and professional achievements in later life are impaired [11]. These children are often underweight and stunted, with higher prevalence in rural than urban children [12]. This also applies for Kenya, where stunting and underweight affect 20% and 7% of urban, and 30% and 13% or rural children, respectively [13]. Urban/rural differences in micronutrient status in Kenyan children are less distinct, nevertheless prevalence or micronutrient deficiency tend to be higher in rural settings [14].

Even though urban/rural differences are investigated, disparities within urban or rural groups are often neglected. Disparities within groups are higher in urban than in rural groups [15], which is challenging for the interpretation of mean values that are usually reported. When comparing prevalence of malnutrition of lowest socioeconomic group for urban and rural settings, prevalence of stunting is still lower in urban than rural children, yet the difference is extremely small [16]. Accordingly, when adjusting for socioeconomic status, urban/rural differences in child malnutrition reported in Demographic and Health Surveys are often not observed [15]. Similar applies to the influence of food insecurity to malnutrition. The prevalence of malnutrition in children increases with food insecurity [17,18], which applies also for the lowest income groups after controlling for wealth status. Wealth status was calculated as a latent variable computed from a composite measure of household assets and amenities [17].

We assumed that malnutrition is more prevalent in food-insecure rural children than in urban ones. This difference demands different strategies for nutrition improvement in the respective settings. The purpose of this study is to describe and compare malnutrition and micronutrient deficiencies linked to dietary intake among urban and rural schoolchildren from food insecure settings in Kenya.

## 2. Materials and Methods

### 2.1. Study Design and Sites

In this paper, analyses of the baseline data from a study on the impact of baobab fruit pulp (*Adansonia digitata L.*) consumption on the iron status (*Baobab Nutrition Intervention Study*) are presented. We selected children with low hemoglobin levels at screening because we expected to see greater improvements in their iron status through the intervention with the additional supply of vitamin C through baobab fruit pulp compared to children without anemia. The number of children was calculated for the *Baobab Nutrition Intervention Study*. Sites were selected according to a reported low prevalence of malaria, high levels of food insecurity, and no or little access to baobab fruits. The public pre-and primary schools were purposely selected according to the following criteria: (a) school meal program in place, (b) day school, (c) about 250 children aged 6–12 years, and (d) accessibility by car. For both schools, we only approached schools were the authorities and the head masters were known to be supportive and open for the intervention. The study was implemented in May 2017 and 2018, respectively towards the end of the rainy season. In 2017, the amount of rain was below normal rainfall while it was above normal in 2018. In both settings, school meals were not fortified.

In 2017, the study was conducted in an urban resource poor setting at Heidemarie Primary School in Mathare IV, (Nairobi, Kenya). About 1400 children attended the urban school, which was supported by the school meal program of the World Food Programme of the United Nations. Families had to pay a fee (11 Kenyan shillings per day, which equals 0.10 USD) at the beginning of each trimester for participation in the school meal program. One year later, a second round of the study was implemented at the rural Kakumuti Primary School in Kakumuti, a village in Musengo zone, Kitui-West, Eastern Province of Kenya, approximately 165 km away from Nairobi. About 430 children attended the school with a self-governed school meal program. At the beginning of the trimester, families had to bring maize and beans, as well as firewood during the trimester.

### 2.2. Sample Size

The sample size calculation was based on the planned *Baobab Nutrition Intervention Study* of which the baseline data are presented here. With a total of 66 children aged 6–12 years for the urban and rural school, respectively, an assumed dropout of 10%, and a prevalence 5% of homozygote forms of sickle cell and α-thalassemia traits or a coincidence of heterozygote forms, data on 56 children was required for a planned *Baobab Nutrition Intervention Study*.

### 2.3. Sampling of Study Participants

**Figure 1:**
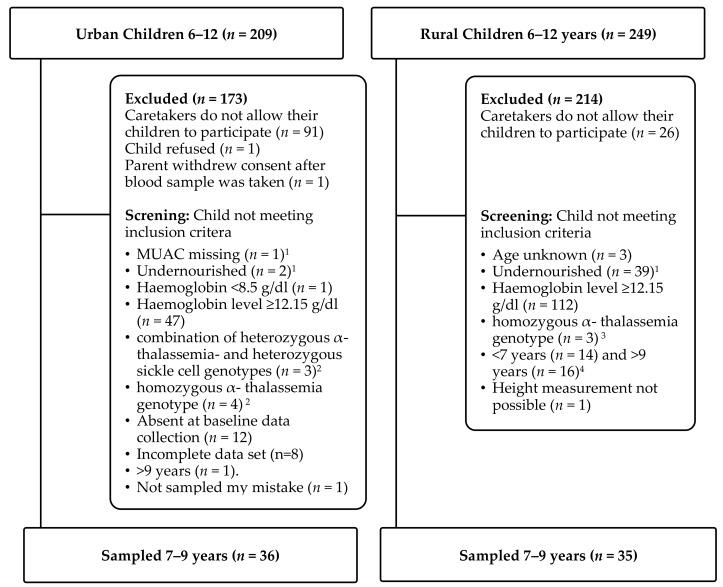
Consort Diagram of the Sampling Procedure. ^1^ Moderate undernutrition defined as mid-upper arm circumference (MUAC) < 14.5 cm for children aged 5–9 years and <18.5 cm for children aged 10–15 years by National AIDS/STI Control Program [21]; ^2^ Blood samples for genotyping collected at screening; ^3^ Blood samples for genotyping collected at baseline; ^4^ Reference dates: 3 May 2017 and 7 May 2018.

After receiving official research permits and consent from the representatives of the purposely selected urban and rural schools, locally trained project assistants described the study in Kiswahili (urban setting) and Kikamba (rural setting) to caregivers of the children eligible for screening. Written informed consent (signature or fingerprint) was obtained from children’s primary caretakers prior to any data collection. Only children of caretakers who gave consent were invited to the screening. The assistants orally informed these children about the study objective and procedure of the upcoming exercise, and children signed a sheet to document that they have been informed. Children’s oral consent and their signature was prerequisite for any further interview and examination. Locally trained nurses and laboratory technicians performed the clinical screening in a separate room and gave all children a deworming remedy (albendazole USP 400). Exclusion criteria are shown in Figure 1.

### 2.4. Questionnaires and 24h-Recalls

Interviewer with a formal background in nutrition or food science, as well as literate in English and the local languages, were trained on applying standardized semi-structured questionnaires and 24h-recalls with primary caregiver. The questionnaire and 24h-recalls used were translated into the local languages (Kiswahili and Kikamba) and retranslated into English, reviewed during the 6-day interview training, pre-tested and modified to ensure meaning equivalence of the questions. Pre-testing was carried out among households with children not sampled for the study.

With the tablet-based questionnaires developed with Open Data Kit (ODK), information on the history of disease during one month preceding the interview, infant feeding, socioeconomic status, water, sanitation and hygiene, and location of the household with GPS was collected. The linear distance from households to the schools was determined with the software ArcGISPro 2.3 (https://www.esri.com/de-de/arcgis/about-arcgis/overview).

The interviews for the multiple-pass 24h-recalls consisted of (a) listing all foods and drinks consumed the day before the interview, (b) gathering detailed information about each food or recipe for dishes, (c) estimated quantification of the amount of consumed food/drink and used ingredients for the recipes, and (d) reviewing the information with the respondent at the end of the recall. Specially designed photo books for each the urban and rural setting were developed to estimate the quantity of intake of food and drinks. Interviewer also used local measuring tools such as spoons and cups for quantifying portion sizes.

Table 1 shows the recommended dietary allowances for energy, vitamins, and trace elements for school-aged children. Individual energy adequacy ratios were calculated as total energy intake divided by sex, and age-specific energy requirements, based on the recommendations of the FAO/WHO/UNU expert committee on human energy requirements [19]. The nutrient adequacy ratio (NAR) was determined for vitamin C, B12, and A (defined as retinol equivalent), as well as for iron and zinc. Individual NARs were calculated as a total intake of the nutrient divided by the recommended daily allowance (RDA) for that nutrient, based on intakes recommended by the Kenyan Ministry of Health [14].

To evaluate the source of nutrients, food and meals consumed were allocated to a modified food group list [20]:

(1) Starchy staple food
  (a) Ugali (stiff cornmeal porridge made from maize flour)
  (b) Chapati (unleavened fried flatbread made from wheat flour)
  (c) Mandazi (African doughnut made from wheat flour)
(2) Mixed dishes with legumes
(3) Vegetables and fruits
  (a) Vitamin A-rich fruits
  (b) Green leafy vegetables
(4) Flesh foods (any kind of meat, organ meat, seafood, insects, and insect eggs)
(5) Dairy products
(6) Eggs
(7) Others (fat, tea, sugar and sweets, spices)

The food group ‘others’ contains ingredients that could only be quantified for overall nutrient intake and is therefore not included in Table 6. As children in the rural setting consumed different types of beans in one mixed dish, while urban children only consumed kidney beans, a food group-mixed dish with legumes was constructed. In the urban setting, it was common to buy cooked beans and add them to mixed dishes, while in the rural setting dried beans were used and boiled as part of the preparation process. Therefore, the total amount of beans per meal could not be compared. For *chapati* and *mandazi*, only the overall nutrient intake could be evaluated.

Mixed dishes with legumes were commonly consumed at home and at school. Table 2 shows the intake from regular school meals, a mixture of maize and beans. The data presented here are baseline data of the *Baobab Nutrition Intervention Study*. Any intervention-related foods and drinks were not included in the data analysis.

### 2.5. Anthropometric Measurements

At screening, registered local nurses received an additional instruction on how to assess the mid-upper arm circumference (MUAC) with a measuring tape that allows an assessment to the nearest 0.1 cm. Moderate undernutrition was defined at MUAC <14.5 cm and <18.5 cm for children aged 6–9 years and 10–12 years, respectively [21]. At baseline, children were checked for edema and weighed without shoes and in light clothing to the nearest 0.1 kg, using a Seca (R) UNICEF scale (mod. 874, SECA, Hamburg, Germany). Body height was measured to the nearest 0.5 cm using a calibrated SECA (R) height scale (SECA 213). Weight and height measurements were repeated twice with a maximum tolerable difference of 0.1 kg for weight and 0.5 cm for height.

The weight-for-age *z*-score (WAZ), body mass index-for-age *z*-score (BAZ), and height-for-age *z*-score (HAZ) were calculated using Anthro Plus, the anthropometric calculator module based on the 2007 WHO reference for children aged 5–19 years [22,23]. Stunting, underweight, and thinness were defined by HAZ, WAZ, and BAZ below—2SD, respectively. The school provided data on the age of the children, which was crosschecked with primary caregivers. If the primary caregiver could not verify the date of birth, WAZ, BAZ, and HAZ were not calculated.

### 2.6. Blood Sample Collection and Analysis

To minimize any discomfort, a local anesthetic ointment containing lidocaine and prilocaine (EMLA^TM^) was applied onto the area of skin to be numbed prior to pricking. At screening, capillary blood samples of children were taken for two subsequent hemoglobin measurements using a HemoCue HB 301 photometer device (HemoCue AB, Ängelholm, Sweden). The maximum tolerated difference between the measurements was 0.5 g/dL. The mean value was used to determine hemoglobin levels.

At baseline, local nurses took from each child a non-fasting venous blood sample, which was spun within 30 min to obtain 50–100 µL serum. The serum was pipetted into labelled 0.2 mL Multiply^®^ PCR tubes (Sarstedt 72.737.002). In the field, samples were either stored cool for a maximum of seven days and then put into a freezer or stored in a freezer on the same day [24]. The samples were taken to the laboratory (VitMin Lab, Willstaett, Germany), where serum ferritin, soluble transferrin receptor (sTfR), retinol-biding proteins (RBP), acidic glycoprotein (AGP), and C-reactive protein (CRP) levels were analyzed using a sandwich ELISA [25]. The Wako zinc test was applied to determine serum zinc via colorimetric measurements [26].

Hemoglobin was adjusted for altitude and anemia defined as adjusted hemoglobin < 11.5 g/dL in children aged 7–11 and <12 g/dL in children aged 12 [27]. Iron deficiency was defined by depleted iron stores (adjusted serum ferritin <15 g/L) [28] and tissue iron deficiency by high serum sTfR (>8.3 mg/L) [25]. RBP concentrations were used as a surrogate measure for circulating retinol to evaluate vitamin A status. Vitamin A deficiency (VAD) was defined by adjusted serum RBP < 0.70 mol/L [29].

CRP and AGP were assessed for identifying and classifying inflammation: incubation (CRP levels > 5 mg/L and AGP levels ≤ 1 g/L), early convalescence (CRP levels > 5 mg/L and AGP levels > 1 g/L), late convalescence (CRP levels ≤ 5 mg/L and AGP levels > 1 g/L). Serum ferritin and RBP concentration were adjusted for inflammation stage with correction factors for each inflammation stage [30,31]. Zinc deficiency without clinical signs was identified by serum zinc < 0.65 µg/dL only [32]. Genotyping for sickle cell trait and the common African 3.7 kb a-globin a+-thalassemia deletion was conducted via PCR [33,34] at KEMRI Wellcome Trust laboratories in Kilifi, Kenya.

### 2.7. Data Management and Statistical Analysis

Data entry and validation via double entry was performed for anthropometry and hemoglobin, as well as for the 24 h recalls. The country-specific food database for Kenya was read into the NutriSurvey nutrient database. Missing food items were supplemented from the Tanzania Food Composition Tables [35] the FoodData Central of the United States Department of Agriculture [36]. Data management and statistical analysis were executed using SPSS software version 24 (IBM Corp., Armonk, NY, USA). Normality of distributions was evaluated using the Shapiro–Wilk test. As most continuous variables (anthropometry, micronutrient status, and nutrient intake) had unequal distributions, descriptive statistics for continual variables are therefore represented by the median, interquartile range (IQR), and minimum and maximum values. The non-parametric median test was applied for comparing data from urban and rural children. The strength of association was calculated with Cramer’s V, which equals r. Variables were tested for associations with non-parametric Spearman’s correlation. A *p*-value of <0.05 was considered statistically significant.

### 2.8. Ethics

The institutional review board of the Faculty of Medicine at Justus Liebig University Giessen, Germany (197/16) and the AMREF Ethics and Scientific Review Committee (AMREF—ESRC P313/2017) Kenya, approved the Baobab Nutrition Intervention Study—which is not further reported here—under the Kenyan National Commission for Science, Technology and Innovation research permit (NACOSTI/P/17/60305/15018 and NACOSTI/P/18/60305/20841). The study was registered with the German Clinical Trials Registry (DRKS00011935). The municipal and governmental authorities approved the implementation of the study in both schools. Written informed consent of primary caregiver and schoolchildren via signature or fingerprint was obtained prior to data collection. The ethics committees also approved the consent format prior to data collection.

## 3. Results

### 3.1. Enrollment of Screened Participants

Figure 1 shows the number of children who participated in the study in the format of a CONSORT diagram. After the screening, the age distribution of eligible children was centered to 7–9 years in the urban group while equally distributed in the rural group. In order to increase homogeneity of the target groups, age was restricted to 7–9 years in both groups for data analysis except for reported underweight and high hemoglobin levels at screening.

### 3.2. Child Care

The majority of children enrolled were girls (urban: 75.0%; rural: 51.4%). Except for one urban child, all children were breastfed, and 63.9% of urban and 60.0% of rural children were initially breastfed within the first hour after delivery. The median duration of exclusive breastfeeding was four months in both settings. Among all children, 27.8% of urban and 32.4% of rural children had been exclusively breastfed for six months. Table 3 shows reported sickness of 30 urban and 34 rural children one month preceding the interview.

The reported prevalence of diseases was similar in both groups except for the lower prevalence of fever in urban children (20.0%, *n* = 30) than in rural (61.8%, *n* = 34) ones. Deworming within the past six months was reported for 73.3% of urban (*n* = 30) and 67.6% of rural (*n* = 34) schoolchildren. Five urban and seven rural respondents remembered to provide a supplement to their children, while only vitamin A supplements were recognized, namely by three and four respondents, respectively.

### 3.3. Nutritional Status

The results of the anthropometric assessment among children are shown in Table 4. At screening, the prevalence of underweight children aged 6–9 years was low in both settings (urban: 0%, *n* = 110; rural: 0.9%, *n* = 112), but much higher in the age group 10–12 years (urban: 33.3%, *n* = 6; rural: 35.2%, *n* = 108). Medians of weight and height of urban children were 2.7 kg and 5.8 cm higher than those of rural children. These differences were significant (weight: χ^2^(1) = 6.222, *p* = 0.013, r = 0.296; height: χ^2^(1) = 4.079, *p* = 0.043, r = 0.240). Regarding moderate underweight (WAZ ≤ −2 SD), wasting (BAZ ≤ −2 SD), and stunting (HAZ ≤ −2 SD), the prevalence was lower in urban children than in rural ones, namely 2.8% vs. 23.5%, 0% vs. 11.8%, and 11.1% vs. 17.7%, respectively. One urban child was found to be overweight (BAZ > 2 SD). The differences in median *z*-scores were significant for underweight and wasting (WAZ: χ^2^(1) = 14.641, *p* < 0.001, r = 0.457; BAZ: χ^2^(1) = 11.209, *p* = 0.001, r = 0.400).

### 3.4. Hemoglobin and Micronutrient Status

Results of the blood analysis are presented in Table 5. At screening, the prevalence of anemia was 28.0% (*n* = 118) in the urban and 13.0% (*n* = 223) in the rural setting. Median adjusted hemoglobin levels differed significantly between groups (χ^2^(1) = 17.643, *p* < 0.001, r = 0.227). After screening children with the lowest hemoglobin level, the anemia prevalence in the study population increased to 38.9% in urban children and 28.6% in rural ones.

The prevalence of iron deficiency was lower in urban than rural children (2.9% vs. 14.3%). Tissue iron deficiency was present in 11.8% of urban and 8.6% of rural children. The prevalence of VAD was higher in urban (14.7%) than rural (8.6%) children. Zinc deficiency was only found in 34.3% of rural children. Furthermore, significantly higher values for adjusted serum ferritin and zinc were found in urban children than in rural ones (ferritin: χ^2^(1) = 6.385, *p* = 0.012, r = 0.304; zinc: χ^2^(1) = 19.871, *p* <0.001, r = 0.537).

The number of children with inflammation stage incubation, early convalescence, and late convalescence was low in both groups, namely 1, 2, and 0 urban children and 1, 0, 3 rural children, respectively. Genotyping for sickle cell and α-thalassemia traits detected two urban children with sickle cell, as well as 12 urban and 13 rural children with α-thalassemia traits. None of these hemoglobinopathies were found among the remaining 22 children in either group.

### 3.5. Dietary Intake

The frequency of consumption and median nutrient intake through certain food groups are shown in Table 6. High frequency of consumption and highest medium intake of energy, iron, and zinc were reported from mixed dishes with legumes. Green leafy vegetables (GLV) were frequently consumed in both groups and contributed mainly to vitamin C and provitamin A intake. Intake of high caloric snacks and food (*mandazi* and *chapati*) were higher in the urban setting than in the rural one.

Urban children had a lower intake of micronutrients than their rural counterparts, apart from provitamin A and Vitamin B12 (Table 7). Overall, dietary intake was below Kenyan RDAs in urban and rural children, except for zinc in the rural setting. The median adequacy ratio for energy was low, with no significant differences between urban and rural children. Median NAR of vitamin C (χ²(1) = 4.016, *p* = 0.045, r = 0.250) iron (χ²(1) = 9.035, *p* = 0.003, r = 0.376) and zinc (χ²(1) = 9.035, *p* < 0.003, r = 0.376) was significantly lower in urban children than in rural ones.

### 3.6. Characteristics of Households

Table 8 shows descriptive characteristics of 36 urban and 33 rural households. The three most common income sources in urban areas were employment, petty trade, and casual labor (temporary worker), while in the rural area, casual labor (temporary worker), sale of agricultural products, and employment were reported. One urban household did not mention any income. Only 2.8% of urban, but 35.3% of rural households, reported generating income from self-produced agricultural commodities.

All households in the rural setting grew maize and pulses. Pumpkin, cassava, sweet potatoes, and sorghum were grown by 78.8%, 33.3%, 30.3%, and 24.2%, respectively. Only 8.8% of the households were dependent on subsistence cropping throughout the year. None of the urban households reported having a home garden with vegetables, access to fruit trees, and/or livestock. Among rural households, only three grew vegetables, but 24 had access to fruit trees. Both vegetables and fruits were mainly used for their own consumption. Conversely, among 24 rural households keeping livestock, one in four used ASF mainly for their own consumption, another one in four mainly for selling, and one in three for both in approximately equal amounts. One in six used livestock mainly for ploughing, transportation, and manure. Median distances between households and schools were 331 m (IQR 98–497 m, min. 39 m, max. 1059 m) in the urban setting and 820 m (IQR 364–1134 m, min. 108 m, max. 2269 m) in the rural setting. The difference was significant (χ2(1) = 10.197, *p* = 0.001, r = 0.431).

## 4. Discussion

Among slight to moderate anemic children in Kenya, we found significantly better nutritional, iron and zinc status in urban than rural children. Whereas energy intake was similar in both groups, iron and zinc intake of urban children was even lower than the intake of rural children.

Although the children in this study were selected with a tendency towards lower hemoglobin levels, another study with Kenyan schoolchildren from urban underprivileged areas reported a similar prevalence of underweight, wasting, and stunting [37], and some even came up with higher rates [38,39]. Notably, the latter studies included children in wider age ranges, namely 5–14 years and 6–12 years, respectively. In rural populations, similar negative *z*-scores were observed in children aged 3–8 years [40,41] and 6–14 years [42], while another study found positive *z*-scores in children aged 7–9 years, except for HAZ in children aged 8 and 9 years [43]. In both settings, the sex of the child, years of education of respondent, and distance to school did not correlate significantly with malnutrition. Overall, stunting was similarly reported in both settings. However, in the urban setting stunted height with predominantly appropriate BAZ indicates a sufficient energy intake, but chronic poor-quality nutrition [44]. Conversely, in rural children, insufficient energy intake and chronically poor-quality nutrition are coexistent. Furthermore, the percentage of caregivers who reported their child to have had fever or a cough in the month preceding the interview was higher in the rural setting. Underweight and wasting is mainly due to a recent weigh loss that may not be attributed to lower food intake only, but also to (infectious) diseases.

Similarly to our findings, a low prevalence of anemia was previously found in underprivileged children in Nairobi [38]; however, in this study, the mean children’s age was higher, and anemia tend to decrease during childhood [45]. Studies in rural settings in Kenya reported a higher prevalence of anemia [41,42], but those studies were conducted in malaria-endemic zones.

Zinc and iron (serum ferritin) statuses were significantly better in urban than rural children while tissue iron stores (sTfR) did not differ. Other studies confirmed a higher prevalence of iron deficiency in underprivileged urban settings (4.8%) [38] than in rural ones (33%, 15%, 6.3%) [40,41,42] in Kenya. However, national data indicated 79.9% schoolchildren with low zinc status, with a lower prevalence in urban children than in rural ones [14].

The finding of median RBP levels around the lower limit of the normal range is not surprising as a poor vitamin A status in children screened for lower hemoglobin levels was expected [46]. Already the Kenyan micronutrient study reported a low VAD prevalence of 3.9% in urban and 5.3% in rural settings [14]. Only one third of the children in both settings met the NAR as intake of provitamin A and preformed vitamin A is low. The number of children with elevated inflammation markers was low and associated neither with adjusted ferritin nor RBP levels.

Heterozygous genotype of α thalassemia was not associated with Hb, ferritin or sTfR levels similar to findings of other investigators, who found this association only for homozygous genotypes in Kenyan preschoolers [41].

The linear distance from households to school was significantly shorter for urban children. Therefore, the physical activity of rural children was likely to be higher, and the higher energy requirement in rural children may contribute to their poorer nutritional status. We did not assess physical activity further; the distance to school was the only estimate. Neither urban nor rural children had access to transportation to and from the school, and in-school physical activity was observed as being very similar.

One food supplementation study over two years found weight and height gain positively predicted by mean daily intake of food energy from ASF containing heme iron, vitamin A, and vitamin B12. Diets high in plant-based foods were associated with poorer growth [47]. Our data supported this observation as intake of flesh foods, dairy products, and eggs was higher in the urban group.

Blood and anthropometric variables indicated a better iron, zinc, and nutritional status in urban children, while rural children had similar NAR for energy, but a higher NAR for iron and zinc than urban children. In both groups, the diets were predominantly based on starchy staple food (mainly maize, wheat, and rice) and legumes (mainly beans) that contributed considerably to the non-heme iron and zinc intake. Several inhibitors (phytate, polyphenols, etc.) reduced the bioavailability of iron and zinc. The higher intake of non-heme iron and zinc in rural children than in urban ones is attributed to a twofold higher intake of mixed dishes with legumes. In the 24 h recalls, there was no report of the use of soda ash to soften maize and beans or to keep the dark green color of green leafy vegetables during cooking, a method that reduces bioavailability of iron and zinc [48]. One reason for the different data on the Hb and iron status may be that the urban group has more access to flesh foods and consumed fewer vegetables. Thereby, anemia due to deficiencies of B-vitamins might be more common in the urban group.

Consumption of heme iron from flesh food was low in both settings; yet, higher in the urban than in the rural group. In agreement, Farber et al. [49] observed higher consumption of flesh foods in urban than rural South African children under <2 years. In our study, none urban household kept livestock or poultry; therefore they were dependent on the market supply. In Nairobi, beef and poultry expenditure rise with income [9,50] and the price was identified the most important reason for not consuming the animal source food (beef meat, eggs, fish, and yoghurt) [51]. Among 24 rural households keeping livestock, 25% used the animal products commercially, which was not observed for plant-based food. In line with our findings, a study from Western Kenya found that most plant-based food produced in rural households were utilized for home consumption (starchy staple food, vegetables, fruits and pulses), while 29% of ASF was sold in markets [52]. In conclusion, the role of ASF for income generations is greater than for plant-based food, which may contribute to a low consumption of ASF in rural settings. Investigators also reported a low consumption of ASF in rural Kitui county in adults [53]. A higher bioavailability of heme iron and zinc from ASF may explain the significantly better iron and zinc status in the urban group.

Among the few studies on diets consumed in underprivileged areas in Nairobi, low energy intake in children was reported [7,41,54], as well as vitamin A intake below Kenyan RDA combined with adequate iron intake [7]. Studies from rural areas showed a diverse picture ranging from approaching recommendations [43] to low intakes of energy, iron, vitamins C and B12, [10,42,55,56], vitamin A [10,42,56], and zinc [10,42]. In this study, consumption frequency and portion sizes of common high-calorie street foods, namely *chapati* and *mandazi*, were higher in urban children than in rural ones. Wheat flour and vegetable oil, the main ingredients for *chapati* and *mandazi*, are fortified with iron, zinc, and vitamins B12 and A in Kenya [57]. Therefore, the urban group may have benefitted from this fortification.

Overall, GLVs were the main source of provitamin A, but in the urban setting, GLV portion sizes were too low to meet the RDA. These children also ate and drank dairy products as an additional source of vitamin A. As none of the urban households cultivates vegetables and fruits nor kept livestock, household’s food demand was fully dependent on market supply and their economic resources. Among rural children, the amount of GLV consumed was usually high and contributed significantly to meet the RDA. The 24 h recalls were conducted when green leafy vegetables were in season, which might be aligned with the high quantity of consumption. In line with a higher consumption of GLV in the rural group, indigenous GLV were found to be higher in rural than urban dwellers [58]. In our study, the frequency of consumption of other provitamin A rich vegetables and fruits was low. Median portion size of milk per day was too small to contribute substantially to vitamin A intake.

The nutrient intake from school meals differed marginal between the groups (see Table 2) and resulted in a slightly higher intake for vitamin C and A for the meals in the urban school. Moreover, the food energy of the urban school meal was higher. However, this did not translate into a higher overall food energy intake for the schoolchildren. Therefore, the differences in dietary intake and micronutrient status are attributed to dietary intake outside of the school. The urban school met the recommendations of the Kenya’s national guideline for healthy diets, which states that meals comprise three to four food groups [59]. Cooked maize and beans were the only components of the rural school meal.

### Limitations

When conducting the 24 h recall, information on fortified products was insufficiently recorded. Therefore, we cannot provide more information on the actual intake of fortified food. Since 2012 Kenya has made fortification of wheat and maize flour (including iron, zinc, vitamin B12, and provitamin A) and vegetable oils and fats (provitamin A) mandatory. Notably, the general consumption of fortified wheat flour has increased in recent years, but the rate is higher in the higher income groups [57], and the school meals were prepared from unprocessed maize. Still, the total micronutrient intake may be underestimated. Furthermore, the level of consumption of fortified products outside school may differ between the urban and rural settings.

Another limitation is the small sample size. The number of children enrolled was determined by the sample size calculation of the *Baobab Nutrition Intervention Study* of which the baseline data are presented here. A full survey including all children might prove the evidence, but as children with Hb > 12.2 g/dL have been excluded in both groups, the comparison appears to be justified.

Overall, the children’s and caregivers’ availability and willingness to participate in the interviews were lower in the urban setting than in the rural one. Likewise, higher response rates in rural settings were observed in the Kenyan Demographic and Health Survey [13]. Urban underprivileged parents are very busy to generate a small income and do not want to spare time for the interviews from which they do not expect any personal benefit.

## 5. Conclusions

The nutritional status of underprivileged urban and rural schoolchildren differs. In the urban setting, the low intake of nutrients is of greater concern than nutrient bioavailability and vice versa in the rural setting. Besides poor vitamin A intake and status in both groups, the better iron and zinc status among urban children implies access to food with higher bioavailability of iron and zinc there, although vitamin B12 intake is far below recommendations in both settings.

Strategies that address a higher intake of iron and zinc may include better access to flesh food and promote preparation practices that increase the bioavailability of non-heme iron and zinc. To address the low intake of vitamin A and vitamin B12, interventions should include more meat, fish, dark green leafy vegetables, and other vegetables and fruits rich in provitamin A.

Up to now, guidelines for school meals address the schoolchildren as one homogenous group. Knowing the urban/rural differences in the needs of the pupils may facilitate specifically adapted guidelines, e.g., more vegetables in urban, and more ASF in rural school meals.

## Figures and Tables

**Table 1:** Recommended dietary allowances of energy, vitamin, and trace elements for school-aged children.

Energy and Nutrients ^1^	7–8 Years	9 Years
Energy (kcal)	1694	1916
Vitamin C (mg)	25	45
Vitamin B12 (µg)	1	1.8
RE (µg)	400	600
Iron (mg)	10	8
Zinc (mg)	5	8

^1^ Reference values of the Kenya National Micronutrient Survey 2011 [14] except for energy (presented as means for boys/girls aged 7–8 years and 9 years, respectively) [19]; RE: retinol equivalent.

**Table 2:** Energy and nutrients of one regular portion of school meal (calculated in NutriSurvey©).

Variables	Urban (Portion = 248 g)	Rural (Portion = 234 g)
Energy (kcal)	312	277
Vitamin C (mg)	5	0
Vitamin B12 (µg)	0.0	0.0
RE (µg)	17	0
Iron (mg)	3.6	3.3
Zinc (mg)	1.6	1.7

**Table 3:** History of diseases among schoolchildren in the month preceding the interview.

Disease	Urban—*n* = 30		Rural—*n* = 34	
	*n*	%	*n*	%
Fever	6	20.0	21	61.8
Diarrhea (watery stools ≥ 3 times/day)	2	6.6	0	0
Stomach upset/aches	11	36.7	13	38.2
Nausea and/or vomiting	4	13.3	8	23.5
Cough	12	40.0	22	64.7
Malaria (positively tested)	0	0	3	8.8
Child took medication, in case of (multiple answers)				
Fever	4	13.3	9	26.5
Diarrhea	3	10.0	1	2.9
Stomach upset/aches	2	6.7	3	8.8
Nausea and/or vomiting	3	10.0	2	5.9
Cough	5	16.7	16	47.1
Malaria	1	3.3	14	41.2

**Table 4:** Median and IQR of anthropometry and nutritional status of schoolchildren.

	Urban			Rural			Median Test
Variables	*n*	Median	IQR	*n*	Median	IQR	*p*
**Screening—MUAC**							
6–9 years	111	17.0	16.4–18.0	112	16.6	15.6–17.7	0.200
10–12 years	6	19.0	17.8–20.0	108	19.0	17.7–20.0	**-**
Missing	1			3			
**Sampled children**							
7–9 years	36	17.0	16.5–18.0	35	16.5	155–18.0	0.268
**Anthropometry**							
Weight (kg)	36	24.8	22.8–27.4	35	22.1	19.7–24.2	0.013
Height (cm)	36	129.4	122.8–131.2	35	123.6	118.5–129.0	0.043
**Nutritional Status**							
WAZ	36	−0.6	−1.2–0.1	34	−1.5	−1.9–−0.8	<0.001
BAZ	36	−0.3	−0.8–0.3	34	−1.0	−1.4–−0.6	0.001
HAZ	36	−0.6	−1.4–0.3	34	−1.1	−1.6–−0.4	0.151

Date of birth was not confirmed for one rural child, and nutritional status was not calculated, BAZ: body mass index-for-age *z*-score; HAZ: height-for-age *z*-score, MUAC: mid-upper arm circumference, WAZ: weight-for-age *z*-score.

**Table 5:** Median and IQR of hemoglobin, iron, vitamin A, zinc status, and subclinical inflammation of schoolchildren.

	Urban			Rural			Median
Variables	*n*	Median	IQR	*n*	Median	IQR	Test
**Screening—Hemoglobin**							***p***
Hb in g/dL	118	12.6	11.9–13.1	223	12.7	12.0–13.3	0.212
Hb g/dL, adjusted	118	12.1	11.4–12.6	223	12.5	11.8–13.1	<0.001
Sampled children							
Hb g/dL, adjusted	36	11.7	11.2–12.0	35	11.8	11.4–12.0	0.712
**Iron, Vitamin A, Zinc and inflammation marker status**							
**Iron status**							
Ferritin µg/L	34	56.9	39.9–114.5	35	39.2	24.0–67.8	0.041
Ferritin µg/L, adjusted	34	56.9	38.5–96.6	35	39.2	24.0–67.8	0.012
sTfR mg/L	34	5.9	5.1-6.7	35	6.2	5.4–6.8	0.398
**Vitamin A status**							
RBP µmol/L	34	0.86	0.74–1.08	35	0.91	0.84–1.05	0.398
RBP µmol/L, adjusted	34	0.88	0.76–1.09	35	0.91	0.84–1.05	0.717
**Zinc status**							
Zinc (µg/dL)	34	84.0	77.3–90.7	35	68.2	63.4–77.7	<0.001
**Inflammation**							
CRP (in mg/L)	34	0.24	0.14–0.89	35	0.19	0.09–0.49	0.548
AGP (in g/L)	34	0.50	0.43–0.70	35	0.48	0.39–0.64	0.548

AGP: acidic glycoprotein, CRP: C-reactive protein, Hb: hemoglobin, RBP: retinol-binding proteins, sTfR: soluble transferrin receptor.

**Table 6:** Frequency (n), median (IQR) of intake in gram, energy, and nutrients from selected food groups of schoolchildren.

		Food Intake	Energy	Vitamin C	Vitamin B12	RE	Iron	Zinc
	*n*	(g)	(kcal)	(mg)	(µg)	(µg)	(mg)	(mg)
**Urban (*n* = 30)**								
Starchy staple food	79	88 (45–181)	192 (117–230)	0 (0–0)	0 (0–0)	0 (0–0)	1.3 (0.3–2.3)	0.8 (0.4–1.2)
*Ugali ^1^*	22	112 (112–112)	162 (129–240)	0 (0–0)	0 (0–0)	0 (0–0)	1.6 (1.2–2.3)	0.9 (0.7–1.3)
*Chapati ^2^*	10	93 (45–135)	219 (110–311)	0 (0–0)	0 (0–0)	0 (0–4)	1.2 (0.8–1.3)	0.8 (0.4–0.9)
*Mandazi ^3^*	20	55 (55–87)	235 (235–370)	0 (0–0)	0 (0–0)	0 (0–0)	0.5 (0.5–0.8)	0.3 (0.3–0.4)
Mixed dishes with legumes	34	248 (103–248)	312 (110–324)	3.1 (0.5–6.3)	0 (0–0)	6 (0–17)	3.6 (1.7–3.8)	1.6 (0.7–1.7)
Vegetables and Fruits	112	13 (6–39)	3 (1–11)	2.2 (0.5–6.1)	0 (0–0)	8 (0–25)	0.1 (0.0–0.3)	0 (0.0–0.1)
Vitamin A rich fruits	2	81.5	23.8	54.7	0	179	0.3	0.05
GLV	22	29 (9–52)	9 (2–15)	7.4 (3.5–20.2)	0.0 (0.0–0.0)	84 (34–305)	0.4 (0.2–0.8)	0.1 (0.0–0.1)
Flesh Food	9	31 (22–56)	94 (45–184)	0.2 (0–5.2)	0.8 (0.1–2.8)	1 (0–7)	0.6 (0.3–2.5)	1.3 (0.5–2.6)
Dairy products	23	60 (46–95)	40 (31–63)	0.6 (0.5–1.0)	0.2 (0.2–0.4)	33 (26–52)	0.1 (0–0.1)	0.2 (0.2–0.4)
Eggs	1	8	12	0	0.1	10	0.1	0.1
**Rural (*n* = 34)**								
Starchy staple food	126	76 (47–142)	169 (142–247)	0 (0–0)	0 (0–0)	0 (0–0)	1.7 (0.5–1.7)	0.8 (0.3–0.9)
*Ugali ^1^*	23	202 (202–202)	271 (221–348)	0 (0–0)	0 (0–0)	0 (0–0)	2.6 (2.1–3.3)	1.5 (1.2–1.8)
*Chapati ^2^*	3	180	466	0	0	0	1.2	0.9
*Mandazi ^3^*	9	50 (28–66)	214 (120–282)	0 (0–0)	0 (0–0)	0 (0–0)	0 (0–0)	0.3 (0.1–0.4)
Mixed dishes with legumes	64	235 (197–235)	277 (277–277)	0.2 (0.2–2.0)	0 (0–0)	0 (0–0)	3.3 (1.9–3.3)	1.7 (1.7–1.7)
Vegetables and Fruits	130	22 (10–59)	6 (3–14)	1.9 (0.5–7.4)	0 (0–0)	12 (0–46)	0.1 (0.1–0.3)	0 (0.0–0.1)
Vitamin A rich fruits	1	133	17	109.1	0	124	0.6	0.2
GLV	19	88 (69–112)	33 (25–41)	11.6 (8.8–21.1)	0.0 (0.0–0.0)	467 (356–599)	0.7 (0.6–1.4)	1.2 (0.9–1.6)
Flesh Food	2	32	87	0	0.6	0	0.6	1.4
Dairy products	33	44 (22–57)	29 (15–38)	0.4 (0.2–0.6)	0.1 (0.1–0.2)	24 (12–31)	0 (0–0.1)	0.2 (0.1–0.2)
Eggs	0							

**^1^***Ugali:* stiff cornmeal porridge, ^2^*Chapati*: unleavened fried flatbread, ^3^*Mandazi*: African doughnut, GLV: green leafy vegetables.

**Table 7:** Median and IQR of adequacy ratio of energy and nutrient intake of schoolchildren in percent.

	Urban *n* = 30		Rural *n* = 34		
	Percentage the NAR Achieved by the Children				Median Test
	Median	IQR	Median	IQR	*p*
Energy	63	45–83	69	56–87	0.316
Vitamin C	49	22–89	65	33–146	0.045
Vitamin B12	25	0–66	10	2–14	0.316
RE	33	10–90	29	17–115	1.000
Iron	77	64–91	96	80–122	0.003
Zinc	71	45–106	109	91–133	0.003

**Table 8:** Descriptive characteristics of households of the schoolchildren enrolled in the study.

	Urban *n* = 36		Rural *n* = 33	
Characteristics	*n*	%	*n*	%
**Sex of respondent**				
Male	2	5.6	3	9.1
Female	34	94.4	30	90.9
**Sex of the head of the household**				
Male	26	72.2	28	84.8
Female	10	27.8	5	15.2
**Level of education of respondent attended**				
No schooling	2	5.6	0	0.0
Some primary	9	25.0	11	33.3
Completed primary (class 8)	12	33.3	17	51.5
Some secondary	2	5.6	3	9.1
Completed secondary (class 12)	10	27.8	2	6.1
More than secondary	1	2.8	0	0.0
**Level of education of head of household**				
*Respondent is head of the HH*	1	2.8	8	24.2
No schooling	0	0.0	0	0.0
Some primary	13	36.1	6	24.0
Completed primary (class 8)	5	13.9	10	40.0
Some secondary	4	11.1	6	24.0
Completed secondary (class 12)	12	33.3	2	8.0
More than secondary	1	2.8	1	4.0
**Reason for settling in Nairobi/Kitui-West**				
Born in this area	2	5.6	7	21.2
Moved here by marriage	7	19.4	26	78.8
Wanted better livelihood	11	30.6	0	0.0
Work in this area	7	19.4	0	0.0
Looking for job opportunity	2	5.6	0	0.0
Other ^1^	7	19.4	0	0.0
**Three main sources of income** (multiple answers)				
Casual labor (temporary worker)	12	33.3	21	61.8
Sale of agricultural products	1	2.8	12	35.3
Employment	19	52.8	3	8.8
Petty trade	15	41.7	5	14.7
Sale of goods/crafts	1	2.8	6	17.6
Remittances from abroad	0	0.0	3	8.8
Other ^2^	6	16.7	1	3.0
None	1	2.8	0	0.0

^1^ Good infrastructure (*n* = 1), cheap rent (*n* = 5), good security (*n* = 1); ^2^ Women’s group (microfinance) (*n* = 1), washing people’s clothes (*n* = 1), not specified (*n* = 5).
